# Radial Peripapillary Capillary Network in Patients with Retinitis Pigmentosa: An Optical Coherence Tomography Angiography Study

**DOI:** 10.3389/fneur.2017.00572

**Published:** 2017-10-27

**Authors:** Rodolfo Mastropasqua, Enrico Borrelli, Luca Agnifili, Lisa Toto, Luca Di Antonio, Alfonso Senatore, Michele Palmieri, Alessandro D’Uffizi, Paolo Carpineto

**Affiliations:** ^1^Moorfields Eye Hospital NHS Foundation Trust, London, United Kingdom; ^2^Ophthalmology Clinic, Department of Medicine and Science of Ageing, University G. D’Annunzio Chieti-Pescara, Chieti, Italy; ^3^Doheny Image Reading Center, Doheny Eye Institute, Los Angeles, CA, United States; ^4^Department of Ophthalmology, David Geffen School of Medicine at UCLA, Los Angeles, CA, United States

**Keywords:** retinitis pigmentosa, optical coherence tomography angiography, optic nerve, retinal nerve fiber layer, vascular density, optical coherence tomography

## Abstract

**Purpose:**

To investigate radial peripapillary capillary (RPC) network in patients affected by retinitis pigmentosa (RP).

**Methods:**

Eleven patients (22 eyes) with previous diagnosis of RP and 16 age-matched healthy subjects (16 eyes) were enrolled. The diagnosis of RP was made based on both clinical features and electrophysiological examination. All patients underwent a complete ophthalmologic examination, including optical coherence tomography angiography and visual field (VF). The primary outcomes were the RPC vessel density in the peripapillary and disk areas; the secondary outcomes were the peripapillary retinal nerve fiber layer (RNFL) thickness and the mean defect at VF.

**Results:**

A total of 19 eyes of 11 RP patients (5 males, 6 females) and 16 eyes of 16 healthy subjects (10 males, 6 females) were included for the analysis. RPC vessel density in the disk area was 46.5 ± 7.1% in the RP group and 45.4 ± 10.6% in the control group (*p* = 0.754). RPC vessel density in the peripapillary area was significantly reduced in the RP group after the comparison with the control group (52.5 ± 5.0 and 57.2 ± 5.1%, respectively, *p* = 0.011). RNFL thickness was 85.9 ± 20.4 μm in the RP group and 104.0 ± 6.4 μm in the control group (*p* = 0.002). RPC vessel density was significantly correlated with RNFL thickness values in RP patients, both in the disk and in the peripapillary area (Rho = 0.599 and *p* = 0.007 in the disk area, Rho = 0.665 and *p* = 0.002 in the peripapillary area, respectively).

**Conclusion:**

We showed that density of RPC is reduced in these patients in the peripapillary area. Moreover, the RPC vessel density correlates with the RNFL thickness.

## Introduction

Retinitis pigmentosa (RP) is the name used for a group of inherited retinal disorders that are characterized by progressive loss of the outer retinal cells, and resulting retinal dysfunction (1–5).

Clinically, RP manifests initially as night blindness (nyctalopia) and is eventually followed by clear visual field (VF) loss (1, 4, 5). Characteristic findings by fundus examination include peripheral pigmented bone spicule-like lesions, retinal arteriolar attenuation, and optic disk pallor (6). RP can be considered a photoreceptor disease; however, there is increasing evidence that the inner retina becomes disorganized, with retinal ganglion cell (RGC) death and retinal nerve fiber layer (RNFL) thinning, following the outer retina damage (7–9). Furthermore, there is abundant evidence showing that vascular changes (e.g., perivascular cuffing, arteriolar attenuation, and reduced ocular blood flow) featured RP and were hypothesized to be part of the pathogenic process (10).

Optical coherence tomography angiography (OCTA) has recently been developed to study retinal and choroidal microvasculature without needing a dye injection (11). Moreover, the OCTA technology has allowed the radial peripapillary capillary (RPC) network to be visualized separately, with a satisfactory short-term and long-term reproducibility (12). The RPC can be considered a unique vascular plexus in the RNFL and has been showed to be highly associated with the RNFL thickness (13, 14).

Several studies recently showed vascular alterations in RP by means of OCTA (15–18). However, these studies did not evaluate the RPC network features in RP patients.

In this cross-sectional study, we investigated the RPC network changes in eyes affected by RP, and we assessed the relationships between vascular structure and RNFL thickness.

## Materials and Methods

### Study Participants

Eleven patients (22 eyes) with a previous diagnosis of either mid- or late-stage RP (19) were consecutively enrolled at the Retina Service of the Ophthalmology Clinic, University G. D’Annunzio of Chieti-Pescara, Italy. The institutional review board approved this study, and all patients gave informed consent to the use of their data. The study adhered to the tenets of the Declaration of Helsinki.

The diagnosis of RP was made based on both the clinical features and the electrophysiological examination [multifocal electroretinogram showing a reduced amplitude of N1 (first negative component) and P1 (first positive component) of the first-order kernel]. Patients were selected with a best-corrected visual acuity (BCVA) of at least 0.8 LogMAR to ensure proper execution of the examinations, intraocular pressure (IOP) <18 mmHg (measurement taken at 9:00 a.m.), central corneal thickness (CCT) (Ultrasound pachimetry, Altair; Optikon 2000, Rome, Italy) ranging from 530 to 570 µm, and VF test without signs indicative of glaucoma.

Exclusion criteria were as follows: (i) aged younger than 18 years old; (ii) evidence of an advanced form of RP (either undetectable ERG or extended macular atrophy) (19); (iii) any retinal dystrophy affecting the patient other than RP; (iv) any history of either maculopathy or ocular vascular disease; (v) ocular hypertension, manifest glaucoma, or any condition increasing the risk of secondary glaucoma (e.g., pigment dispersion syndrome or pseudo-exfoliation syndrome); (vi) history of non glaucomatous optic neuropathy (e.g., anterior ischemic optic neuropathy or compressive optic neuropathy); (vii) myopia greater than −3.00 diopters; and (viii) any systemic or topical therapy that could potentially affect retinal and optic disk vascular parameters, or any variation in the last 3 months.

All patients had undergone a complete ophthalmologic examination, which included the measurement of BCVA, IOP (Goldmann applanation tonometry), VF test [Humphrey field analyzer II 750 (Carl Zeiss Meditec Inc., Dublin, CA, USA) (30-2 test, full threshold)], dilated fundus examination, and OCTA.

A healthy control group, homogeneous for age and gender, was also included in the current analysis. All control subjects also underwent a complete ophthalmologic examination, including BCVA, IOP, CCT, VF test, dilated fundus examination, and OCTA.

### Procedures

#### Spectral Domain-OCTA with XR Avanti^®^

The OCT angiography imaging, as well as the RNFL measurement, was made with the XR Avanti^®^ AngioVue OCTA (Optovue Inc., Fremont, CA, USA). This is a device with a high speed of 70,000 axial scans per second using a light source of 840 nm and an axial resolution of 5 µm. The AngioVue OCTA system, which is based on split-spectrum amplitude-decorrelation angiography algorithm (Version: 2016.100.0.45), uses blood flow as an intrinsic contrast. Indeed, the flow is detected as a variation over time in the speckle pattern formed by the interference of light scattered from red blood cells and adjacent tissue structure (20).

The detailed description of disk and peripapillary angioflow vessel density measurement technique has been published elsewhere (21–23). In brief, study participants underwent imaging following a protocol that included the AngioVue OCT 3D volume set of 4.5 mm × 4.5 mm. An internal fixation light was used to center the scanning area. The OCT signal position and quality were optimized by means of the “Auto All” function, which performs in sequence with (i) the “Auto Z” function to find the best position for obtaining the retina OCT image; (ii) the “Auto F” function to find the best focus for the particular subject’s refraction; and (iii) the “Auto P” function to find the best polarization match for the particular subject’s ocular polarization. Then, one FastX (horizontal raster) set and one FastY (vertical raster) set were performed for each acquisition scan. The software then performed the motion correction technology to remove saccades and minor loss of fixation. Scans with low quality (i.e., if the subject blinked or if there were many motion artifacts in the data set) were excluded, and repeated until good quality was achieved. Three scans for each patient were captured, then the best one for quality (without significant motion artifacts and with a signal strength index >60) was considered for the analysis.

#### Vascular Layer Segmentation and Vessel Density Analysis

**Figure 1:**
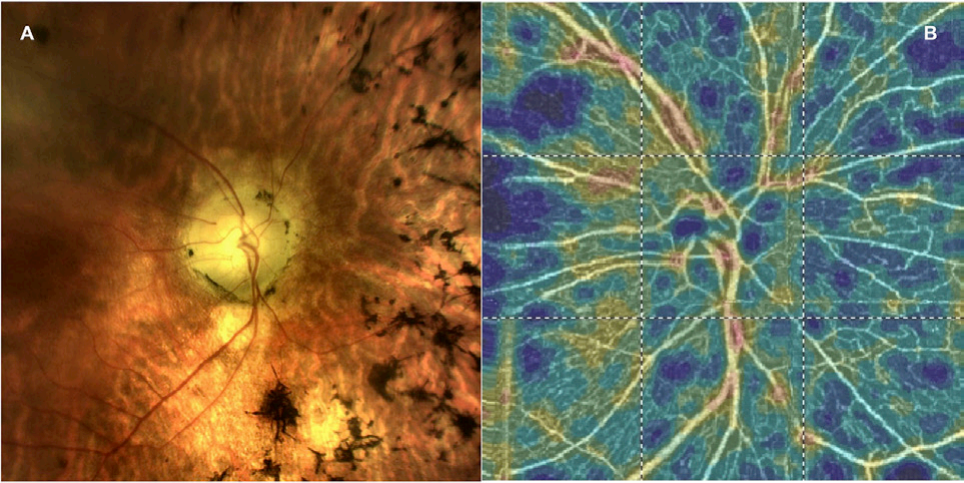
**(A)** A picture of the fundus in a patient with retinitis pigmentosa and **(B)** the corresponding optical coherence tomography angiography 4.5 × 4.5 optic nerve scan showing the radial peripapillary capillary network.

**Figure 2:**
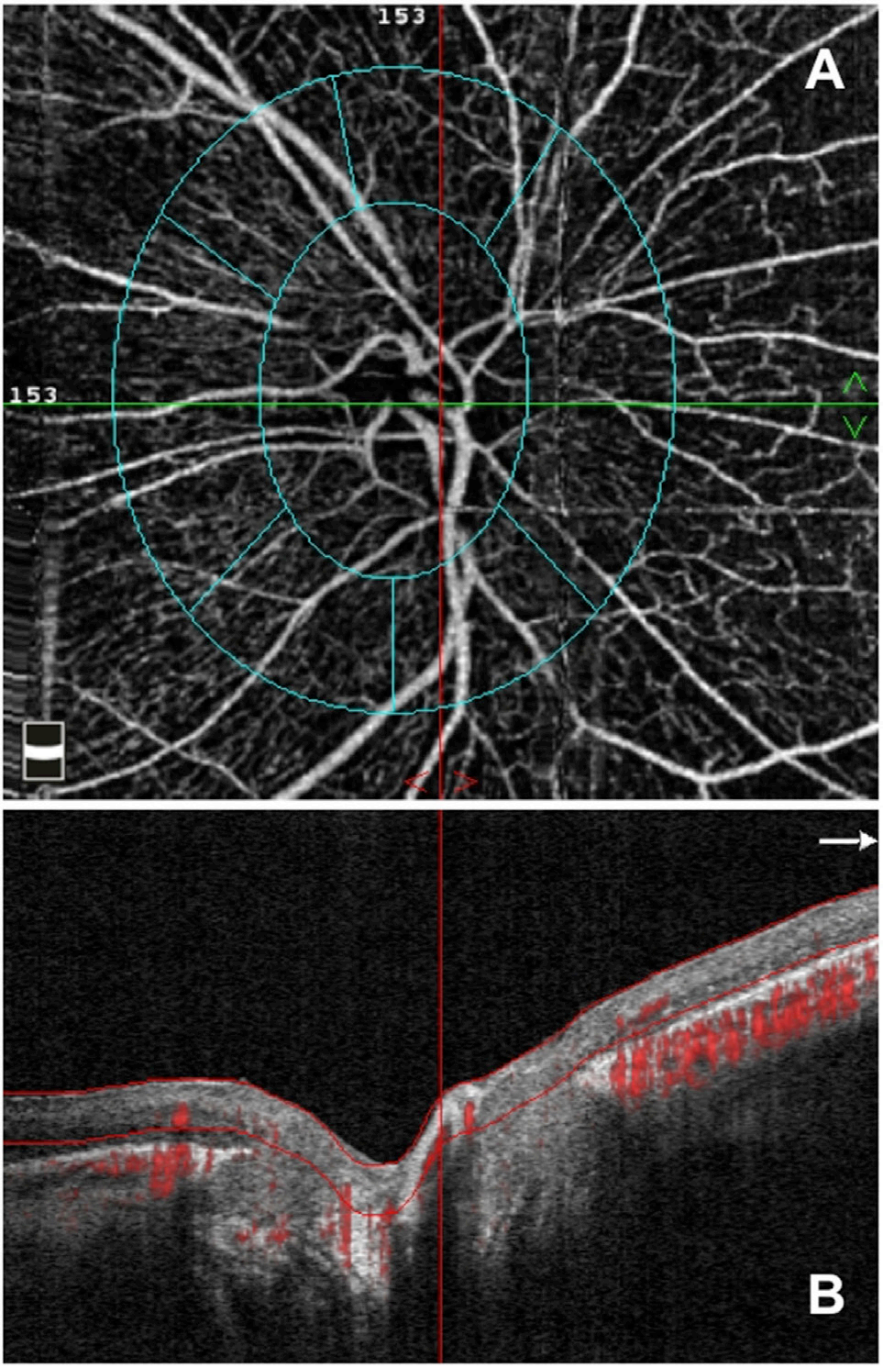
Optical coherence tomography angiography (OCTA) from an enrolled retinitis pigmentosa patient. **(A)** OCTA 4.5 × 4.5 optic nerve scan showing the radial peripapillary capillary (RPC) network and **(B)** OCT B-scan showing the slab set to evaluate the RPC network.

To evaluate the RPC layer, we used a slab between the outer limit of the RNFL and the internal limiting membrane, as previously described (Figures 1 and 2) (13, 14, 24). Two investigators checked and manually adjusted the slab’s segmentation quality before testing the vessel density.

The vessel density was defined as the percentage area occupied by vessels in a region of interest (ROI). The two regions in which the vessel density was tested were as follows: (i) disk area and (ii) peripapillary area, corresponding to the ring between two elliptical contour lines and with a width of 0.75 mm. The inner elliptical contour (which defines the optic nerve head) is obtained by automatic fitting an ellipse to the disk margin based on the OCT en face image. The AngioVue software automatically outputs the flow area value within the two selected ROI.

The primary outcomes were the RPC vessel density in the peripapillary and disk areas; the secondary outcomes were the peripapillary RNFL thickness and the mean defect (MD) at VF.

### Statistical Analysis

All quantitative variables were summarized as mean and SD. To detect departures from normality distribution, the Shapiro–Wilk’s test was performed for all quantitative variables.

The analysis of variance (ANOVA), with nested design, was performed to assess the differences between groups for RNFL thickness and vessel density. The nested ANOVA was used to account for using both eyes from RP patients in the same sample. The false discovery rate (FDR) correction was used to control the family-wise type I error rate, and an FDR adjusted *p*-value < 0.05 was determined to be statistically significant.

Spearman’s correlation coefficient was tested to evaluate the linear correlation among variables in RP patients.

All statistical analyses were performed using Statistical Package for Social Sciences (version 20.0, SPSS Inc., Chicago, IL, USA).

## Results

A total of 19 eyes of 11 RP patients (5 males, 6 females) and 16 eyes of 16 healthy subjects (10 males, 6 females) were included for the analysis. Three eyes of three patients had to be excluded from the analysis because of the inability to obtain scans of enough quality.

Mean age was 40.1 ± 7.3 years (range 23–45 years) for RP patients and 42.2 ± 6.5 years (range 48–76 years) for healthy subjects (*p* = 0.578). BCVA was 0.5 ± 0.1 and 0.0 ± 0.0 LogMAR in the RP and control groups, respectively (*p* < 0.0001).

All the subjects enrolled were Caucasian and not affected by diabetes or glaucoma. Furthermore, there were no significant differences in systemic hypertension or the use of systemic antihypertensive medications between the groups.

Mean IOP and mean CCT were 17.4 ± 2.5 and 16.7 ± 2.1 mmHg and 549 ± 11.2 and 558 ± 15.3 μm, in RP and control subjects, respectively (*p* > 0.05 for both parameters).

Radial peripapillary capillary vessel density in the disk area was 46.5 ± 7.1% in the RP group and 45.4 ± 10.6% in the control group (*p* = 0.754). RPC vessel density in the peripapillary area was significantly reduced in the RP group after the comparison with the control group (52.5 ± 5.0 and 57.2 ± 5.1%, respectively, *p* = 0.011).

Retinal nerve fiber layer thickness was 85.9 ± 20.4 μm in the RP group and 104.0 ± 6.4 μm in the control group (*p* = 0.002). RPC vessel density was significantly correlated with RNFL thickness values in RP patients, both in the disk and in the peripapillary area (Rho = 0.599 and *p* = 0.007 in the disk area, Rho = 0.665 and *p* = 0.002 in the peripapillary area, respectively).

Mean defect significantly differed between RP and control subjects, with values of −29.2 ± 1.4 and +1.9 ± 0.6, respectively (*p* < 0.001). Spearman correlation analysis found significant negative correlations between MD and both peripapillary and disk RPC vessel densities (Rho = −0.644 and *p* = 0.004, Rho = −0.875 and *p* < 0.0001), as well as between MD and RNFL thickness (Rho = −0.995 and *p* < 0.0001).

## Discussion

In this cross-sectional study, we investigated the RPC vessel density in patients affected by retinitis pigmentosa. Overall, we found that RP patients were characterized by RPC alterations, and that these alterations were associated with the RNFL thickness.

The RPC network is a unique vascular plexus in the RNFL and has recently been studied by means of OCTA in healthy subjects, as well as in glaucoma patients. Mase et al. (14) quantitatively analyzed the features of RPC visualized using wide-field montage OCTA in healthy human eyes. In the latter study, the RPC vessel density was shown to be highest in the peripapillary region, and to gradually decrease toward the macula. Moreover, healthy subjects were shown to have a significant direct correlation between RPC vessel density and RNFL thickness. A direct correlation between these two parameters, as well as a reduction in peripapillary RPC vessel density, was found in glaucoma patients (13).

Alterations in the ocular blood flow, as well as in the retinal vessels, have been fully described as being part of the RP pathogenesis. Wolf et al. (25) showed that increased arteriovenous transit time and reduced blood flow velocity are early hemodynamic findings of RP patients without any clinically detectable sign of disease. Furthermore, reduction of the retinal blood vessels, with perivascular pigment deposits, is a funduscopic feature of patients affected by RP (4). Our group has recently shown that both choroid and retinal vessels were decreased in RP patients, after comparison with healthy subjects; and that the superficial and deep capillary plexuses vessel densities were correlated with the macular function, evaluated by means of electroretinography (16).

To the best of our knowledge, no study evaluated the RPC network in patients with RP.

Our study first shows that peripapillary RPC is significantly reduced in RP eyes.

In recent years, there has been a debate on the efficacy of photoreceptor restorative therapies. Nevertheless, restoration of vision is only possible if retino-cortical conduction is preserved (26). It is well known that after photoreceptor damage some changes affect inner retinal layers, including RNFL, which was reported thinner in patients affected with RP (27). Some authors have attributed this loss to the reduced trans-synaptic signal following photoreceptor cell degeneration (8, 9). Nevertheless, other authors have implicated diminished blood flow to the inner retinal layers (28). In our study, the RNFL thickness was reduced RP patients, and a direct correlation was present between the RNFL thickness and the RPC vessel densities. The correlation between RNFL thinning and the reduction of blood flow in the RPC might be explained by a reduction of metabolic demand and a subsequent reduction in vascular flow. In addition, the significant correlations between the MD and both angioflow parameters suggest that vascular changes in the disk and peripapillary regions may cooperate with the RGC and RNFL loss in the reduction of the VF sensibility. A decreased vessel density significantly associated with the severity of VF damage was demonstrated in glaucomatous eyes, and this association seems to be independent of the structural loss (29). Nevertheless, given that MD significantly correlated also with RNFL thickness, we cannot ascertain what is the real impact of vascular changes in the final VF loss.

Vascular depletion is thought to be an early event in RP, which eventually causes ischemia and tissue loss. Histopathological studies showed that the reduced ocular blood flow is a primary event due to retinal vessel damage. In fact, vessel narrowing and sclerosis, followed by thickening of the blood vessel wall and, consequently, lumen occlusion, are features of RP (4, 28). Moreover, Konieczka et al. (10) demonstrated that typical symptoms of the primary vascular dysregulation syndrome—an illness characterized by vessel predisposition that reacts differently to a number of stimuli—may occur in RP patients.

In the assumption that vessel damage is an early event, the damage involving the RPC could contribute to RNFL thinning. Nevertheless, the cross-sectional nature of this study cannot us to clarify the cause-and-effect relationship between vessel damage and other alterations. Furthermore, we enrolled patients affected by either mild or late RP stage, thus we are not able to assume that vascular changes are an early event of the disease. However, this is a stimulating field of research, since further prospective OCTA studies in patients with RP could reveal the timing for vascular changes appearance, in relation to the appearance of RNFL and RGC loss, and VF changes.

Our study has some limitations. The series presented here is relatively small. The size of the patients’ group did not let us to differentiate patients’ characteristics by disease stage. However, one should look at the current series in consideration of: (i) the rarity of the RP disease and (ii) the strict inclusion criteria for patients and the control group, as well as the similarity of groups with respect to meaningful characteristics, such as age. In addition, patients had poor genetic characterization and the results can be interpreted only as generic for RP. Finally, since we did not include patients with undetectable ERG and macular atrophy, our results might not be generalized to the advanced stages of RP.

In conclusion, we have provided a fully integrated study of RPC vessels in RP patients and showed that the density of these capillaries is reduced in these patients in the peripapillary area. Moreover, we demonstrated that the RPC vessel density correlates with the RNFL thickness, as well as with MD at VF. Prospective longitudinal studies will help shed further light on the modality of appearance and progression of the peripapillary and disk vessels loss in RP, and their role in the induction of the visual disability. On these bases, in the near future, optic nerve angioflow measures could be considered as potential new useful tools to early detect the onset and progression of RP, and monitor disease activity and efficacy of new therapeutic approaches. Finally, the influence of the vascular status on the efficacy of the photoreceptor restorative therapies should be evaluated.

## Ethics Statement

This study was carried out in accordance with the recommendations of University G. D’Annunzio IRB with written informed consent from all subjects. All subjects gave written informed consent in accordance with the Declaration of Helsinki. The protocol was approved by the University G. D’Annunzio IRB.

## Author Contributions

Study concept and design: RM, EB, and PC. Acquisition, analysis, or interpretation of data; critical revision of the manuscript for important intellectual content: all the authors. Drafting of the manuscript: RM, EB, LA, LT, and LDA. Statistical analysis: EB. Study supervision: PC.

## Conflict of Interest Statement

The authors declare that the research was conducted in the absence of any commercial or financial relationships that could be construed as a potential conflict of interest.
